# Religious Participation and Trajectories of Cognitive Decline in African Americans: The Moderating Effect of Age

**DOI:** 10.1093/geroni/igaf122.436

**Published:** 2025-12-31

**Authors:** Ann Nguyen, Elissa Kim, Weidi Qin, Yoonkyung Shin, Lisa Barnes

**Affiliations:** Case Western Reserve University, Cleveland, Ohio, United States; Wayne State University, Detroit, Michigan, United States; University of Wisconsin–Madison, Madison, Wisconsin, United States; Case Western Reserve University, Cleveland, Ohio, United States; Rush University Medical Center, Chicago, Illinois, United States

## Abstract

African Americans are disproportionately impacted by dementia compared to other racial groups. Religion holds special meaning and significance for African Americans and is an important psychosocial resource that can protect against cognitive decline. The salubrious effects of religion tend to be greater for older adults than younger adults. However, very few studies have examined the connection between religious participation and cognitive decline specifically in African Americans, and none have investigated age differences in this connection. The aims of this study were 1) to determine whether non-organizational religious participation is associated with cognitive status trajectories in this population and 2) to determine whether the association between non-organizational religious participation and cognitive status trajectories vary by age. Data for this study come from the Minority Aging Research Study (N = 845). Non-organizational religious participation measures included religious coping and prayer/meditation. We utilized group-based trajectory modeling to identify cognitive status trajectories based on global cognitive function scores. We identified four distinct cognitive status trajectories: very low and declining; low and declining; moderate and slow decline; and high and stable. While religious participation was not directly related to cognitive status trajectories, the interactions between the religious participation variables and age were significant. The protective effects of religious participation were strongest among old-old participants. Among the oldest-old participants, more frequent religious participation was associated with increased risk for cognitive decline. These findings indicate that the relationship between non-organizational religious participation and cognitive status trajectories is dependent on age. Implications for clinical practice and interventions will be discussed.

